# The effect of perceived busyness on nostalgic consumption intention: based on compensatory control theory

**DOI:** 10.3389/fpsyg.2026.1722695

**Published:** 2026-03-18

**Authors:** Ershuai Huang, Xiaoping Zhu, Feng Yao, Taiyang Zhao

**Affiliations:** 1 Research Center of Energy Economic, School of Business Administration, Henan Polytechnic University, Jiaozuo, China; 2 School of Business and Management, Jilin University, Changchun, China; 3 School of Philosophy and Sociology, Jilin University, Changchun, China

**Keywords:** compensatory control theory, nostalgic consumption, perceived busyness, perceived autonomous, sense of control

## Abstract

**Introduction:**

In modern society, where busyness serves as the main theme, busyness not only affects consumers’ social cognition, physical and mental health, but also influences their behaviors. Previous studies have mostly explored its impact on consumer behavior from the perspective of positive psychological perceptions induced by busyness, with relatively few studies examining its impact from the perspective of negative psychological perceptions triggered by busyness. Based on compensatory control theory, this study aimed to investigate the effect of perceived busyness on nostalgic consumption, as well as the mediating role of the sense of control and the moderating role of perceived autonomy.

**Methods:**

We collected data from 655 participants across four studies. Data were analyzed using statistical techniques including t-tests, linear regression, and mediation and moderation effect analyses.

**Results:**

The findings revealed that: (1) perceived busyness positively influences consumers’ willingness to engage in nostalgic consumption; (2) sense of control mediates this relationship; and (3) perceived autonomy moderates both the direct effect of busyness on nostalgic consumption and the mediating effect of sense of control, confirming a pattern of moderated mediation.

**Discussion:**

This research expands the theoretical understanding of perceived busyness within consumer behavior. It also provides practical insights for policymakers and organizers regarding mental health management for individuals experiencing high levels of busyness.

## 1 Introduction

In contemporary society, characterized by information explosion and accelerated technological change, continuous busyness has gradually become the norm for individuals worldwide. According to the International Labor Organization, in 2023, more than one-third of the world’s workers worked more than 48 h per week, far exceeding the internationally accepted standard of 40 h per week. Behind this widespread phenomenon lies the systemic stress triggered by intensified global competition and economic uncertainty. Whether it is organizations adhering to the “efficiency first” logic to maintain competitiveness or individuals being compelled to devote more working hours to cope with rising living costs, extending working hours has become a widely adopted coping strategy in economies across multiple countries. In the rapidly developing regions of East Asia, Southeast Asia, and the Pacific, the proportion of workers with a weekly working time exceeding 48 h is notably higher than the global average (International Labour Organization, 2023), which further highlights the regional intensity of this trend. The high pace of work has a direct impact on public mental health. The 2024 report of Gallup’s State of the World’s Workplace indicates that 41% of employees globally are in long-term high-pressure situations, with 52% of employees in the Middle East and North Africa reporting being under significant pressure. On the global social platform Instagram alone, there are over 12 million posts under the hashtag “Burnout,” with the cumulative number of interactions (likes, comments, shares) far exceeding 10 billion, further reflecting the widespread public anxiety about this high-intensity lifestyle. This anxiety does not stay at the level of social discussion but directly translates into structural changes in the consumer market. For individuals facing difficulty in breaking away from the logic of efficiency-based survival in reality, consumer behavior is an important outlet for releasing pressure, regulating emotions, and achieving psychological compensation (Bagwell and Bernheim, 1996; Williams, 2014). Therefore, exploring the impact of perceived busyness on consumer behavior holds certain practical significance.

Previous studies have shown that perceived busyness affects individuals’ social cognition (Bellezza et al., 2017; Ebrahimi et al., 2017; Gershuny, 2005, 2009; Kim et al., 2019; Malika and Maheswaran, 2023) and physical and mental health (Caruso, 2014; Gershuny, 2003; Kleppa et al., 2008; Pulkki-Råback et al., 2012; Van Dongen and Belenky, 2009). However, few studies have examined the effect of perceived busyness on consumer behavior (Linh and Rhee, 2020; Yang et al., 2024). Moreover, most existing studies have focused on the effects of positive psychological perceptions triggered by perceived busyness on consumer behavior. For instance, the sense of perceived busyness increases consumers’ perceptions of self-importance, leading to impulsive rather than indulgent consumption of hedonic products (Linh and Rhee, 2020). Furthermore, the sense of busyness enhances consumers’ perceptions of self-efficacy, making them receptive to new products (Yang et al., 2024). However, perceived busyness is likely to induce negative psychological perceptions in individuals. Perceived busyness is accompanied by negative emotions, such as anger (Hsee et al., 2008), anxiety, and depression (Virtanen et al., 2012), which induce negative psychological perceptions, such as self-threatening perceptions (Bekker et al., 2003), resource scarcity perceptions (Bruch and Ghoshal, 2002), and control deprivation (Whitson and Galinsky, 2008). Nevertheless, few studies have explored the impact of perceived busyness on consumer behavior from the perspective of negative psychological perceptions triggered by it.

This study explores the impact of perceived busyness on consumer behavior from the perspective of a lack of control. Social psychology research suggests that humans inherently have a desire to exert control over their environment, and acquiring and maintaining a high level of personal control is a fundamental human need (Averill, 1973). When a busy work and life routine becomes the norm for individuals, the cognitive load (Pontari and Schlenker, 2000; Wilcox et al., 2016) and negative emotions (Raghunathan and Pham, 1999; Virtanen et al., 2012) brought about by a high-intensity work pace and heavy workload can reduce their ability to cope with environmental changes. This, in turn, leads to a cognitive perception of uncertainty about their current and near-future states (Milliken, 1987), ultimately resulting in a negative psychological perception of a lack of control (Bos, 2007; Lachman and Weaver, 1998).

According to compensatory control theory, when consumers’ sense of control is threatened, they have a strong motivation to restore it (Kay et al., 2009; Landau et al., 2015). Nostalgic consumption, as a carrier of the “past,” evokes memories of a known, complete, and unchangeable past. The past is highly certain, authentic, and predictable within an individual’s narrative (Tester, 2002), allowing individuals to temporarily escape from the present, filled with uncertainties, and restore their sense of control over the external environment and their own lives. Meanwhile, nostalgia, as a highly accessible and everyday emotional experience (Newman et al., 2020), does not require excessive cognitive effort, making it a low-threshold and highly efficient means of psychological compensation. So, can nostalgic consumption repair the negative psychological perception induced by perceived busyness? Few studies have explored the relationship between the two. Thus, this paper will examine the impact of perceived busyness on nostalgic consumption, uncover its explanatory mechanism, and identify its external boundary conditions.

Therefore, this study, grounded in compensatory control theory, explores the impact of perceived busyness on nostalgic consumption through four experiments. It reveals the mediating role of sense of control between the two and identifies the boundary conditions of perceived autonomy. By focusing on consumption, this study examines how negative psychological perceptions triggered by perceived busyness influence nostalgic consumption. This not only enriches the existing research in the field of perceived busyness but also helps us to understand how consumers regulate negative psychological perceptions in their fast-paced, high-pressure daily routines. Additionally, it provides management insights for organizations and policymakers on how to alleviate the negative psychological consequences for individuals and guide them in self-adjustment.

## 2 Literature review and hypotheses development

### 2.1 The impact of perceived busyness on nostalgic consumption

Perceived busyness refers to an individual’s subjective psychological state of experiencing time scarcity, heavy tasks, and a lack of autonomous control over time (Bellezza et al., 2017; Gershuny, 2005). Perceived busyness is not merely a state of time pressure; it systematically shapes an individual’s cognitive evaluations of themselves and their environment. High-intensity busyness means that individuals need to handle multiple tasks within a limited time, cope with frequent interruptions and task-switching, leading to the depletion of their cognitive resources. This, in turn, impairs the depth of information processing and the quality of judgment, making their assessments of task progress, their own abilities, and future outcomes vague and unpredictable (Pontari and Schlenker, 2000; Wilcox et al., 2016), thereby subjectively reinforcing a sense of uncertainty (Milliken, 1987). Meanwhile, the continuous depletion of cognitive resources due to perceived busyness weakens the psychological resources individuals use to process complex information and respond to challenges (Raghunathan and Pham, 1999; Virtanen et al., 2012). This reduces their ability to cope with environmental changes and amplifies their vulnerability and anxiety toward uncertainty (Hamilton et al., 2019).

When individuals develop a cognitive perception of uncertainty due to perceived busyness, it triggers their self-regulatory motivation for certainty, aiming to restore inner order and stability (Van den Bos, 2009a,b). Nostalgic consumption can satisfy this need for certainty by evoking individuals’ nostalgic experiences. On one hand, the past that nostalgia refers to, having already occurred, inherently possesses characteristics of high certainty, authenticity, and predictability (Tester, 2002). Regardless of how the external environment changes, memories of the past are essentially stable. On the other hand, the recollections of the past evoked by nostalgic consumption are often idealized memories (Davis, 1979), which can promote positive psychological states (Cheung et al., 2013; Muehling et al., 2004; Weingarten and Wei, 2023; Zhou et al., 2012) and a sense of social connection (Wildschut et al., 2010; Zhou et al., 2008) in individuals. This allows individuals to temporarily detach from the present filled with uncertainty and anchor themselves in a past perceived as simpler, more authentic, and more idealized, thereby alleviating the perception of uncertainty triggered by perceived busyness. Finally, the study by Bi (2024) also demonstrated the mitigating effect of nostalgic consumption on uncertainty.

Since people generally feel busy in daily life, the means to restore certainty should be easily accessible and not require excessive effort. Nostalgia, as a common and everyday emotion, is widely applicable in the marketing of goods and services (Newman et al., 2020). It does not require people to expend too much cognitive effort, making it a highly accessible and effective means to restore the perception of uncertainty caused by perceived busyness. Therefore, the following hypothesis was proposed:

*H1:* Perceived busyness has a positive effect on nostalgic consumption intentions.

### 2.2 The mediating role of the sense of control

The sense of control refers to an individual’s cognition and feeling of being able to control external objects and the environment. Maintaining a sense of control has long been considered a primary driving force in people’s lives (Whitson and Galinsky, 2008; Wyness, 2019). As a fundamental human need, the sense of control serves as an important psychological mechanism for individuals to resist stress, distress, and anxiety caused by the randomness and unpredictability of the environment (Krystal, 1993; Tullett et al., 2015), and is closely related to individual health and wellbeing (Luck et al., 1999). The core of the sense of control lies in whether individuals believe they can influence events and the environment to achieve desired outcomes (Landau et al., 2015; Locke, 1997). The feeling of time scarcity that accompanies perceived busyness can make individuals feel overwhelmed by numerous tasks and activities, leading to a loss of control over their schedules (Du et al., 2024; Roxburgh, 2004). Consequently, their ability to plan, choose, and adjust the pace of their behaviors is severely restricted (Mullainathan and Shafir, 2013), resulting in a decline in their sense of control (Striberger et al., 2023). Meanwhile, being continuously exposed to a high-intensity work pace and heavy workload can impose cognitive load (Pontari and Schlenker, 2000; Wilcox et al., 2016) and negative emotions (Raghunathan and Pham, 1999; Virtanen et al., 2012) on individuals, reducing their ability to cope with environmental changes. This leads to a cognitive perception of uncertainty about whether they can achieve desired outcomes through their efforts (Milliken, 1987), ultimately resulting in a negative psychological perception of a lack of control (Bos, 2007; Lachman and Weaver, 1998; Landau et al., 2015).

Compensatory control theory suggests that when faced with a lack of control, individuals will engage in a series of compensatory behaviors to restore their sense of control, specifically manifesting as a tendency to seek order. This means they show a preference and need for physical or abstract things that are orderly, certain, and predictable (Kay et al., 2009; Landau et al., 2015). For example, participants experiencing a lack of control prefer a certain work environment over an ambiguous one (Ma and Kay, 2017) and are more inclined toward products that provide order and controllability in their lives (Lembregts and Pandelaere, 2019; Linwan and Jiangmeng, 2021; Shepherd et al., 2011). Existing research has also shown that when individuals perceive a lack of control, nostalgic consumption can serve as a compensatory mechanism to address this psychological deficit (Huang et al., 2023). Nostalgic consumption enhances self-continuity by connecting a stable and positive past self with the present and providing life meaning, thereby conceptually reconstructing an individual’s internal order (Hong et al., 2022; Raghunathan and Pham, 1999) and ultimately compensating for the lack of control. Additionally, the nostalgic emotions induced by nostalgic consumption are profound social emotions. During nostalgia, individuals re-establish intimate relationships with others, and these positive relationships enhance the predictability and manageability expectations of receiving more social support when facing future difficulties (Cheung et al., 2013; Weingarten and Wei, 2023), effectively compensating for their lack of control. Therefore, the following hypothesis was proposed:

*H2:* Sense of control mediates the effect of perceived busyness on consumers’ nostalgic consumption intention. Specifically, individuals with high levels of perceived busyness have a more severe sense of control deficit and a higher intention to consume nostalgically than those with low levels of perceived busyness.

### 2.3 The moderating role of perceived autonomy

Autonomy, as one of the core concepts in self-determination theory, refers to the extent to which individuals perceive their choices and behaviors as free, that is, the degree to which they feel their actions stem from their own will rather than external pressure (Chirkov et al., 2005; Deci and Ryan, 2000). Individuals with high autonomy are more inclined to act in accordance with their own values and interests and are less influenced by external norms or expectations (Warren and Campbell, 2014). A lack of autonomy can reduce people’s task performance, especially for tasks that require flexibility, creativity, or complex abilities (Ryan and Deci, 2006). In research related to the consumption field, perceived autonomy not only directly affects consumers’ emotional experiences and behavioral engagement (Ryan and Deci, 2006; Trepte and Reinecke, 2011) but is also closely related to an individual’s sense of control (Inesi et al., 2011), thereby modulating consumers’ compensatory consumption mechanisms.

According to self-determination theory, the fulfillment of autonomy helps enhance individuals’ perception of the causal relationship between their behaviors and outcomes, thereby strengthening their intrinsic sense of control (Langer, 1975; Zuckerman et al., 1978). Specifically, a high level of perceived autonomy, on one hand, implies that individuals have more choices and decision-making freedom, indicating a higher perception of their ability to control the external environment. On the other hand, it can also manifest as individuals’ behaviors being driven by their intrinsic values, making consumers feel that they are engaging in activities they truly want to do and can influence the outcomes of events through their own actions, thus reinforcing their sense of mastery over life (Botti et al., 2009; Botti and McGill, 2011). Therefore, perceived autonomy can serve as an important internal supporting factor for an individual’s sense of control.

Following these lines of thought, this paper posits that perceived autonomy influences the sense of control deficit caused by busyness, which in turn affects nostalgic consumption willingness. Individuals with high perceived autonomy tend to attribute the behaviors that lead to their busyness to self-determined outcomes (such as actively pursuing career growth or personal skill enhancement). They believe that the results of events are influenced by their own actions and are related to their efforts, thereby weakening the sense of control deficit caused by busyness and reducing their willingness to engage in nostalgic consumption. Conversely, individuals with low perceived autonomy are more inclined to attribute the behaviors that result in their busyness to external constraints (such as societal pressures or avoiding shame). They perceive that the outcomes of events are influenced by uncontrollable external factors, rather than solely by their own actions. This, in turn, undermines their ability to control their life situations, amplifies their perception of a lack of control, and ultimately leads to an increased willingness for nostalgic consumption. Therefore, the following hypothesis was proposed:

*H3:* Perceived autonomy moderates the mediating role of the sense of control between perceived busyness and nostalgic consumption. Specifically, a high sense of busyness decreases the sense of control deficit and nostalgic consumption intentions among consumers with high perceived autonomy, whereas it increases the sense of control deficit and nostalgic consumption intentions among consumers with low perceived autonomy.

**FIGURE 1:**
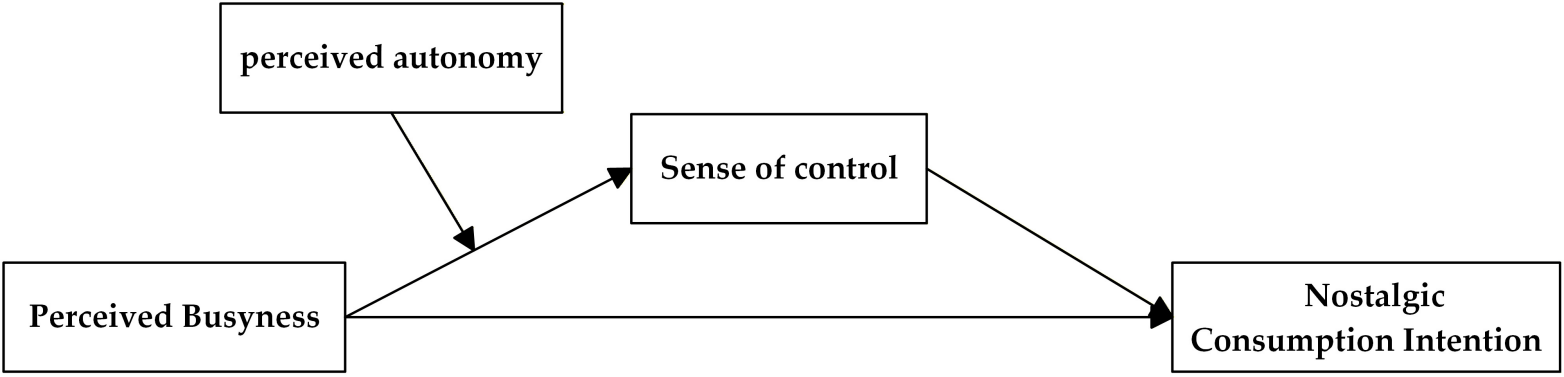
Theoretical model.

Based on the above hypothesis, the theoretical model is presented in Figure 1.

## 3 Materials and methods

We tested our hypotheses in four studies. These studies aim to examine whether situational busyness and chronic busyness influence individuals’ nostalgic consumption intentions through experimental methods. The core hypothesis posits that individuals with busyness (vs. control) will exhibit a stronger intention for nostalgic consumption. Study 1 demonstrated the effect of busyness on nostalgic consumption intention. Study 2 measured chronic busyness to further enhance the generalizability of the effect of busyness on nostalgic consumption intention. Study 3 confirmed the mechanisms—sense of control—that underlie the core effect. Furthermore, Study 4 identified the boundary condition in which the observed effect will be attenuated or intensified by testing the moderating role of perceived autonomy.

### 3.1 Study 1

Study 1 aimed to provide the initial evidence for the influence of perceived busyness on nostalgic consumption intention (H1).

#### 3.1.1 Participants and procedure

Study 1 used a one-way between-subjects design (perceived busyness: busyness group vs. control group). We recruited 174 participants from the Credamo Seeing Numbers platform, an online participant recruitment platform where individuals sign up to complete tasks for money.

The manipulation of perceived busyness was used in a previous research design by asking participants to recall recent experiences in which they perceived busyness (Kim et al., 2019). The participants in the busyness group were asked to recall and write down three recent things that made them feel very busy at work or school, whereas the participants in the control group were asked to recall and write down three things that they often do in their daily lives. After the perceived busyness manipulation, the participants were asked to answer the manipulation test items for busyness with three items (“Right now, I feel very busy.” “Right now, I feel like I have a lot to do.” “Right now, I feel like I don’t have enough time”). Responses were rated on a five-point Likert scale (1 = strongly disagree, 5 = strongly agree). In the present study, the Cronbach’s alpha was 0.93.

Furthermore, nostalgic consumption intentions were measured. The participants were provided with information about two concerts and told to imagine that they received a free concert ticket. They could choose between Concert A (non-nostalgic) and Concert B (nostalgic). The description of the nostalgic concert is designed with reference to the nostalgic appeal of the previous study (Chark, 2022). The theme of Concert A was “Wandering in the Sea of Sound,” and the tagline was “Capture the jumping notes and weave colorful music.” The theme of Concert B was “The Sound of the Years,” and the tagline was “Relive the songs in your memory and tell the story of the passage of time.” To enhance the participants’ sense of immersion in the situation, the reading materials were accompanied by pictures of the corresponding concert tickets. Subsequently, we asked the participants to answer two questions: “Concert A is more nostalgic” and “Concert B is more nostalgic” to test the manipulative effect of nostalgic consumption (5-point Likert scale, 1 = strongly disagree, 5 = strongly agree). Next, the participants’ nostalgic consumption intentions were measured using the following question: “After reading the above information, which concert would you choose to redeem?” Responses were rated on a seven-point Likert scale (1 = strongly prefer concert A, 7 = strongly prefer concert B).

Finally, participants indicated their demographic information (gender, age, and average monthly expenditure) and were paid.

#### 3.1.2 Results

##### 3.1.2.1 Manipulation checks

The independent samples *t*-test revealed that the participants in the busyness group perceived significantly more business than those in the control group [M_*Busyness*_ = 4.40 vs. M_*Control*_ = 2.97; *t*(172) = 10.440, *p* < 0.001], indicating that the busyness manipulation was successful. The paired samples *t*-test demonstrated that the participants reported significantly less nostalgia for Concert A than Concert B [M_*A Concert*_ = 1.72 vs. M_*B Concert*_ = 4.61; *t*(173) = −31.43, *p* < 0.001], indicating that the nostalgia manipulation was successful.

##### 3.1.2.2 Main effects

**FIGURE 2:**
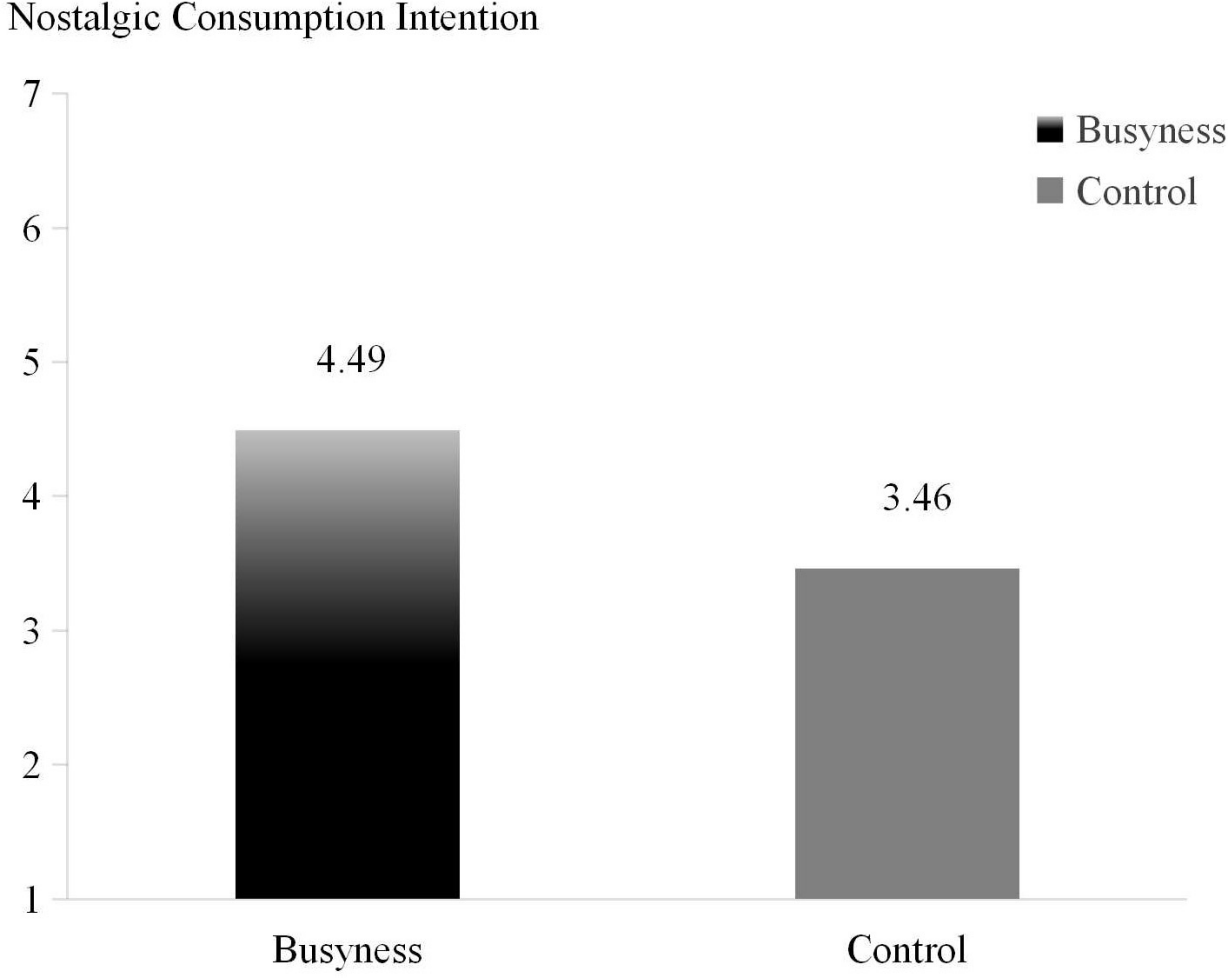
The effect of busyness on nostalgic consumption intention (Study 1).

The independent samples *t*-test used the busyness grouping as the independent variable and the participants’ preference for concerts as the dependent variable, showed that the participants in the busyness group had a significantly higher intention to spend on nostalgic items than those in the control group (M_*Busyness*_ = 4.49, M_*Control*_ = 3.46; *F* = 3.124, *p* < 0.001), supporting H1. A schematic of the main effects was created to show the effect of perceived busyness on nostalgic consumption intention (Figure 2).

#### 3.1.3 Discussion

The results of Study 1 preliminarily demonstrated that situational busyness increased nostalgic consumption. Considering the transient evoked nature of situational busyness in the experimental context, the influence of chronic busyness, which has long existed in daily life, on nostalgic consumption should be examined to explore the external validity of this effect. Thus, Study 2 examined the relationship between chronic busyness and nostalgic consumption intention.

### 3.2 Study 2

Study 2 was to further enhance the generalizability and universality of the effect of perceived busyness on nostalgic consumption intention by measuring chronic busyness.

#### 3.2.1 Participants and procedure

An online questionnaire survey was administered via the Credamo platform. As seven participants failed the attention check, 183 participants (Mage = 28.30, SDage = 7.19) were included in the ensuing analyses.

Perceived busyness was measured using a seven-item scale adapted from Festini et al. (2019). Example items were “I am busy every day,” “I have a lot of things to do every day,” and “I am often rush from one place to another to accomplish something.” Responses were rated on a five-point Likert scale (1 = strongly disagree, 5 = strongly agree). In the present study, the Cronbach’s alpha was 0.861.

Nostalgic consumption intentions were measured by asking the participants to look at two pictures of restaurants (Gu, 2021). Restaurant A was nostalgic with decorations referencing the classroom style of the 1980s and 1990s, whereas Restaurant B was modern with modern decorations. The participants were told that both restaurants featured hot pot and skewers, had close ratings on Dazhong Dianping—China’s dominant crowdsourced review platform for local consumer services, had good word-of-mouth, and had almost the same per capita spending. The participants were asked, “If you wanted to eat hot pot skewers, what restaurant would you be more inclined to visit?” Responses were rated on a seven-point Likert scale (1 = strongly prefer Restaurant A; 7 = strongly prefer Restaurant B).

Finally, participants indicated their demographic information (gender, age, and average monthly expenditure) and were paid.

#### 3.2.2 Results

Linear regression analysis was conducted, with perceived busyness as the independent variable, nostalgic consumption intention as the dependent variable, and gender, age, and average monthly expenditure as the control variables. The results showed that higher levels of perceived busyness significantly predicted greater nostalgic consumption intention (β = −0.243, *p* < 0.001), further supporting H1. See Table 1 for full model statistics.

**TABLE 1:** Results of linear regression analysis for hypothesis testing.

Variable	Nostalgic consumption intention	
	Model 1	Model 2
Gender	0.017	0.021
Age	−0.01	0.008
Average monthly expenditure	−0.01	0.005
Perceived busyness		−0.243***
Adjusted *R*^2^	−0.016	0.038
*F*-value	0.035	2.794*

*p < 0.05,

**p < 0.01,

***p < 0.001.

#### 3.2.3 Discussion

Study 2 reconfirmed H1 by measuring chronic perceived busyness and revealed that perceived busyness significantly predicted stronger nostalgic consumption intentions. The results of Studies 1 and 2 indicated that both chronic and situational perceived busyness have a positive effect on the participants’ nostalgic consumption intention.

### 3.3 Study 3

Study 3 was to examine the mediating role of the sense of control in the relationship between perceived busyness and nostalgic consumption intention (H2).

#### 3.3.1 Participants and procedure

Study 3 used a one-way between-subjects design (perceived busyness: busyness group vs. Control group). We recruited 156 participants from the Credamo Seeing Numbers website platform (M_*age*_ = 30.08, SD_*age*_ = 9.24). After indicating their agreement to participate in this study, participants were randomly assigned to either the busyness group or control group to complete the manipulation task of busyness.

Perceived busyness was manipulated following Kim et al. (2019) procedure used in Study 1. Participants completed the same busyness manipulation task: those in the busyness group recalled and described three recent experiences inducing high busyness at work/school, while the control group recalled three routine daily activities. Following the manipulation, participants answered a busyness manipulation check item: “What is your current level of busyness?” Responses were rated on a five-point Likert scale (1 = very unbusyness, 5 = very busyness).

Subsequently, sense of control was measured using Lachman and Weaver’s 3-item scale (Lachman and Weaver, 1998): “I often feel helpless when dealing with some of life’s problems” “Things that happen in life are often out of my control” “There are many things in life that get in the way of what I want to do.” Responses were rated on a five-point Likert scale (1 = strongly disagree, 5 = strongly agree). In the present study, the Cronbach’s alpha was 0.74.

Furthermore, nostalgic consumption intentions were assessed using the identical concert scenario from Study 1. Participants imagined receiving a free ticket and choosing between Concert A (non-nostalgic): “Wandering in the Sea of Sound” (Tagline: “Capture the jumping notes and weave colorful music”) and Concert B (nostalgic): “The Sound of the Years” (Tagline: “Relive the songs in your memory and tell the story of the passage of time”). To reinforce immersion, concert ticket images accompanied descriptions. Participants then answered two manipulative check questions: “I think Concert A is nostalgic” and “I think Concert B is nostalgic.” “Responses were rated on a five-point Likert scale (1 = strongly disagree, 5 = strongly agree). Finally, nostalgic consumption intention was measured with an item: “Which concert would you prefer to redeem?” Responses were rated on a seven-point Likert scale (1 = strongly prefer concert A, 7 = strongly prefer concert B).

Upon completion, participants provided demographic information (gender, age, and average monthly expenditure) and received compensation.

#### 3.3.2 Results

##### 3.3.2.1 Manipulation checks

The independent samples *t*-test revealed that the participants in the busyness group perceived significantly more business than those in the control group [M_*Busyness*_ = 4.56 vs. M_*Control*_ = 3.42; t (154) = −8.746, *p* < 0.001], indicating that the perceived busyness manipulation was successful. The paired-samples *t*-test demonstrated that the participants reported significantly less nostalgia for Concert A than Concert B [M_*A Concerts*_ = 1.58 vs. M_*B Concerts*_ = 4.63; *t*(155) = −43.832, *p* < 0.001], indicating that the nostalgia manipulation was successful.

##### 3.3.2.2 Main effect

**FIGURE 3:**
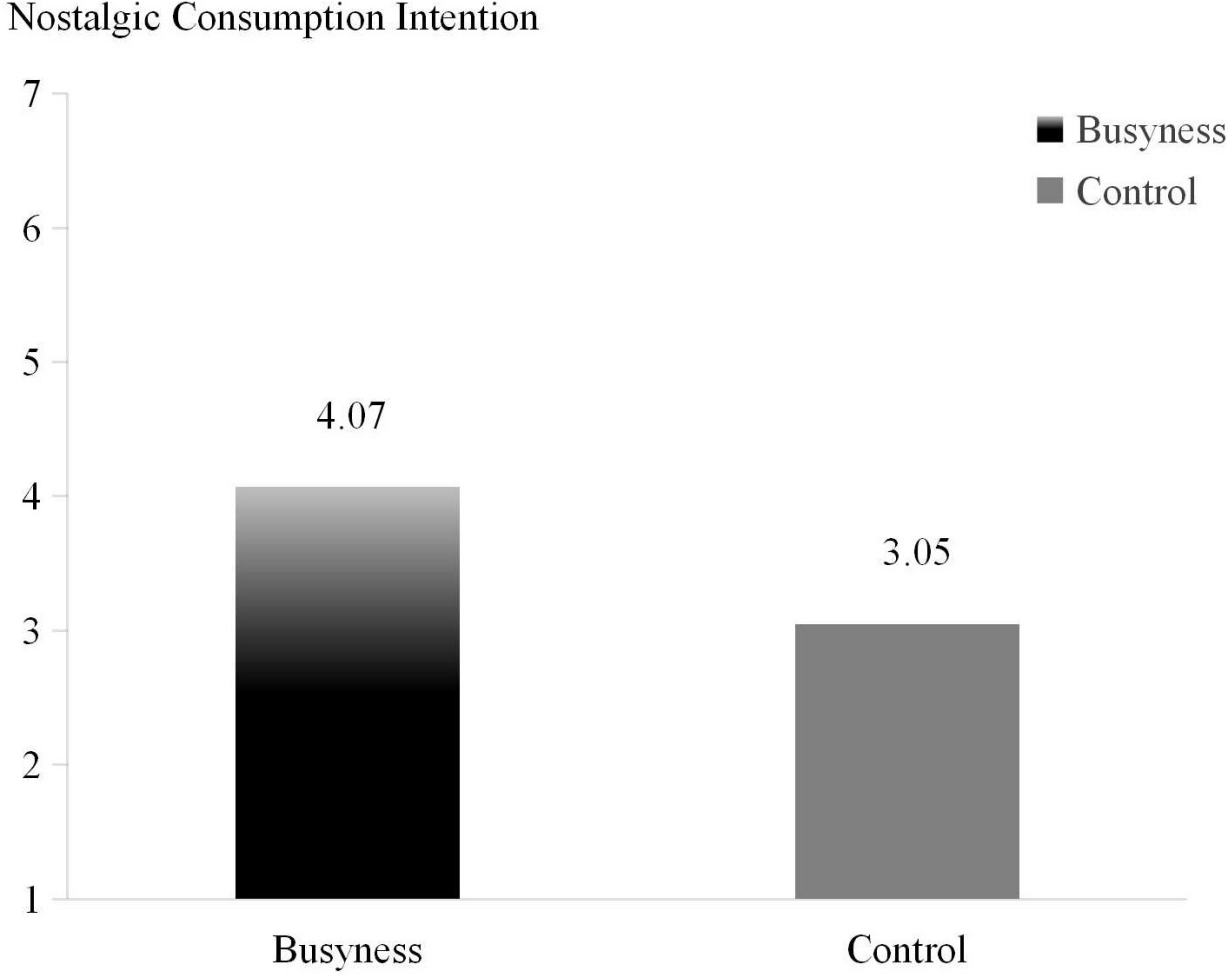
The Effect of busyness on nostalgic consumption intention (Study 3).

The independent samples *t*-test used the busyness grouping as the independent variable and the participants’ preference for concerts as the dependent variable, showed that the participants in the busyness group had a significantly higher intention to spend on nostalgic items than those in the control group [M_*busyness*_ = 4.07, M_*control*_ = 3.05; *t*(154) = −2.826, *p* < 0.001], supporting H1. In this study, a main effects schematic was drawn (see Figure 3) to further show the effect of busyness on nostalgic spending intention.

##### 3.3.2.3 Mediation effect

**FIGURE 4:**
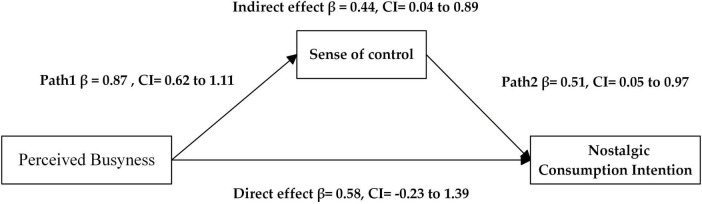
Path analysis results of the model (Study 3).

The bootstrap method was used to test the mediating effect of the sense of control, with perceived busyness as the independent variable, nostalgic consumption intention as the dependent variable, and the sense of control as the mediator variable. We used Process, Model 4, with a confidence interval of 95%. The results revealed a significant mediating effect of the sense of control [Effect = 0.44, SE = 0.22, CI: (0.04, 0.89), not including 0], supporting H2. The results of the model path analysis are presented in Figure 4.

#### 3.3.3 Discussion

Study 3 demonstrated that perceived busyness decreased the participants’ sense of control, leading to their preference for nostalgic consumption. Subsequently, Study 4 explored the boundary conditions external to this psychological mechanism.

### 3.4 Study 4

Study 4 examined the moderating role of perceived autonomy in the relationship between perceived busyness and nostalgic consumption intention. Specifically, high perceived autonomy would weaken the lack of control caused by perceived busyness and reduce consumers’ intention for nostalgic consumption. In contrast, low perceived autonomy increased the lack of control caused by perceived busyness by reducing individual autonomy, thereby increasing consumers’ intention for nostalgic consumption.

#### 3.4.1 Participants and procedure

Study 4 used a one-way between-subjects design (perceived busyness: busyness group vs. Control group). We recruited 142 participants from the Credamo Seeing Numbers website platform (M_*age*_ = 32.56, SD_*age*_ = 6.52). After indicating their agreement to participate in this study, participants were randomly assigned to either the busyness or control group to complete the manipulation task of busyness. Participants first completed the same busyness manipulation procedure as in Study 1. Next, the sense of control was measured following the scale design used in Study 3.

Further, the participants’ perceived autonomy was measured. According to the continuum model of self-determination, the “Relative Autonomy Index (RAI),” as used by Deci, Connell, and Ryan in their 1989 study, is employed to predict participants’ perceived autonomy (Deci et al., 1989). The scale is adapted from Guay et al.’s (2000) Situational Motivation Scale and includes four types of motivation: intrinsic motivation, identified regulation, introjected regulation, and external regulation. These motivation types vary along the underlying dimensions of autonomy support, corresponding to high autonomy, moderate autonomy, moderate control, and high control, respectively. They are assigned weights of + 2, + 1, −1, and −2 to form a composite score reflecting an individual’s level of perceived autonomy. The scale comprises 12 items, such as “I like doing these things” (intrinsic motivation, α = 0.90), “I think these things are meaningful to me” (identified regulation, α = 0.77), “I would feel ashamed or guilty if I didn’t do these things” (introjected regulation, α = 0.70), and “I have no choice but to do these things” (external regulation, α = 0.85). This weighting pattern has two important features: (a) it positively weights autonomy support and negatively weights behavioral control; (b) it assigns heavier weights to stronger instances compared to weaker instances in each situation. Therefore, a high composite score indicates a higher level of perceived autonomy in busy situations, while a low (or negative) score indicates a lower level of perceived autonomy.

Then, nostalgic consumption intentions were measured by asking the participants to choose a camera (Lasaleta et al., 2014). The following introductory statement was used for this scenario: “You need to purchase a camera to record your life. The following two cameras are the same in terms of configuration, performance, and selling price; however, they differ in terms of design concepts and promotional focus.” The participants were presented with promotional images of Cameras A and B. The design concept of Camera A (nostalgic) was “a mark of time, retaining our beautiful past,” whereas the design concept of Camera B (non-nostalgic) was “future vision, a new intelligent camera with one lens.” Participants answered two manipulative check questions: “I think Camera A is nostalgic” and “I think Camera B is nostalgic.” Responses were rated on a five-point Likert scale (1 = strongly disagree, 5 = strongly agree). Nostalgic consumption intention was then measured with an item: “Which camera would you prefer to choose?” Responses were rated on a seven-point Likert scale (1 = strongly prefer Camera A, 7 = strongly prefer Camera B).

Finally, participants indicated their demographic information (gender, age, and average monthly expenditure) and were paid.

#### 3.4.2 Results

##### 3.4.2.1 Rigor of the relative autonomy index method

To verify the rigor and applicability of the Relative Autonomy Index (RAI) in the data of this study by weighing the comprehensive score, the correlation matrices of the four motivation subscales were analyzed (see Table 2). The results provided strong support for the theoretical structure: there was a significant positive correlation between the two types of autonomous motivation (intrinsic motivation and identified regulation) (*r* = 0.641, *p* < 0.001); a significant positive correlation was also found between the two types of controlled motivation (introjected regulation and external regulation) (*r* = 0.453, *p* < 0.001); generally, autonomous motivation and controlled motivation showed negative correlations, especially a strong negative correlation between intrinsic motivation and external regulation, which represent the two ends of the continuum (*r* = −0.677, *p* < 0.001). The above results indicate that the motivational structure measured in this study conforms to the continuum model of self-determination theory, which provides empirical support for the synthesis of a single RAI score using a standard weighting formula.

**TABLE 2:** Descriptive statistics and correlation coefficient matrix of motivation subscales.

Motivator scale	M	SD	1	2	3	4
1. Intrinsic motivation	3.409	1.084	–	–	–	–
2. Identified regulation	3.967	0.773	0.641**			
3. Introjected regulation	3.042	0.966	−0.243**	0.166*		
4. External regulation	3.078	1.089	−0.677**	−0.346**	0.453**	

*p < 0.05,

**p < 0.01,

***p < 0.001.

Notably, a weak positive correlation between identity regulation and ingestion regulation was observed in this study (*r* = 0.166, *p* < 0.05), which may reflect the motivational characteristics of the sample’s cultural context. In East Asian cultures, which emphasize relationships and responsibilities, the individual’s sense of meaning in behavior is often intertwined with the fulfillment of responsibility to others or the collective, forming a kind of “relational autonomy” (Kitayama and Markus, 1991; Li et al., 2015). Therefore, the subjects may regard certain behaviors out of responsibility as actions that are meaningful to themselves. This mixed state of motivation causes the two modes to show a slight co-directional change statistically, but this does not shake the theoretical structure of the continuum in which autonomous motivation and controlled motivation are inversely opposed (Deci and Ryan, 2000; Howard et al., 2020).

##### 3.4.2.2 Manipulation checks

The independent samples *t*-test revealed that the participants in the busyness group perceived significantly more business than those in the control group [M_*busyness*_ = 4.48 vs. M_*control*_ = 3.13; *t*(140) = −9.504, *p* < 0.001], indicating that the perceived busyness manipulation was successful. The paired-samples *t*-test demonstrated that the participants reported significantly less nostalgia for Camera A than Camera B [M_*A Camera*_ = 1.45 vs. M_*B Camera*_ = 4.69; *t*(141) = −32.753, *p* < 0.001], indicating that the nostalgia manipulation was successful.

##### 3.4.2.3 Main effect

**FIGURE 5:**
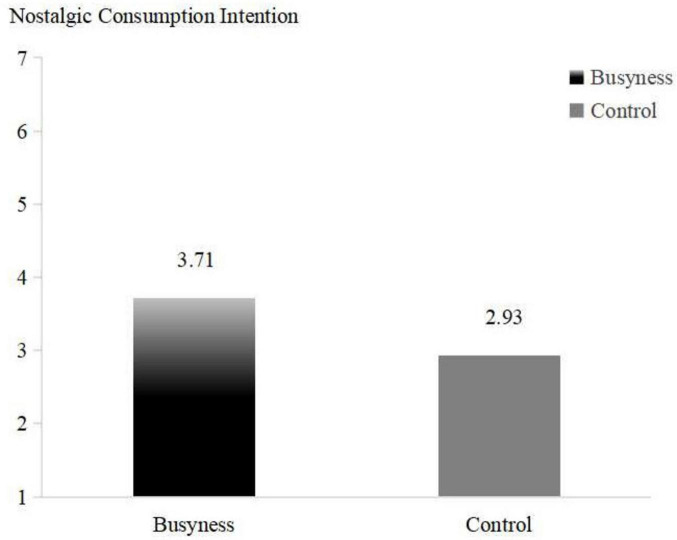
The Effect of busyness on nostalgic consumption intention (Study 4).

The independent samples *t*-test used the busyness grouping as the independent variable and the participants’ preference for cameras as the dependent variable, showed that the participants in the busyness group had a significantly higher intentions to spend on nostalgic items than those in the control group [M_*Busyness*_ = 3.71, M_*Control*_ = 2.93; *t*(140) = −2.009, *p* < 0.05], supporting H1 (see Figure 5).

##### 3.4.2.4 Mediation effect

**FIGURE 6:**
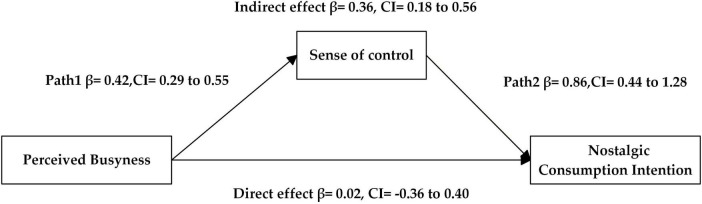
Path analysis results of the model (Study 4).

The Bootstrap method in Model 4 was used to test the mediating effect of sense of control between the effects of perceived busyness on nostalgic consumption intention. With nostalgic consumption intention as the dependent variable, perceived busyness as the independent variable, and sense of control as the mediating variable, the results showed that the mediating effect of sense of control was significant [Effect = 0.36, SE = 0.18, 95% CI: (0.18, 0.56), not including 0], and H2 was established. The results of the model path analysis are presented in Figure 6.

##### 3.4.2.5 The moderated mediation effect

**FIGURE 7:**
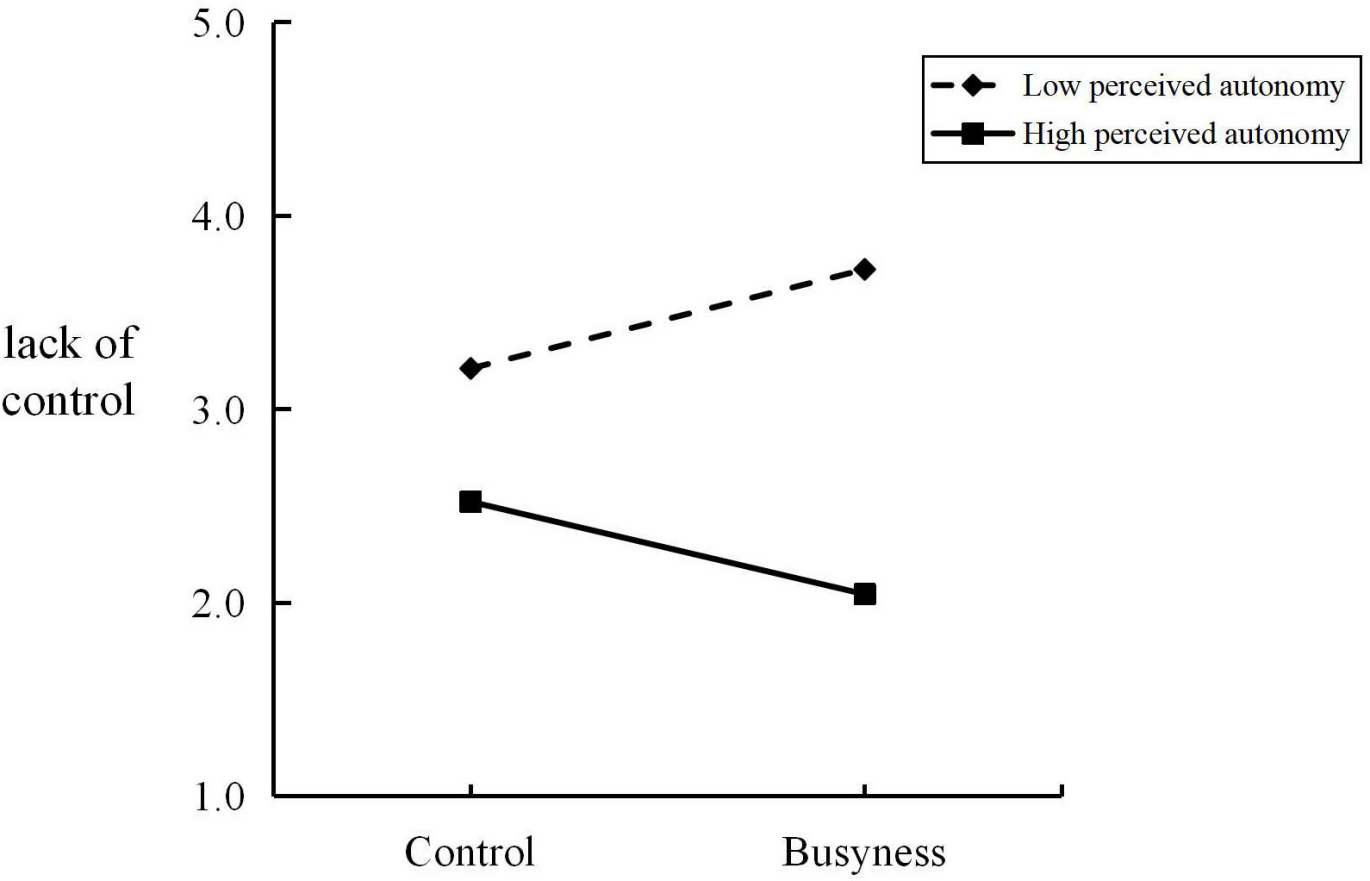
Simple slope plot (Study 4).

The Bootstrap method (Model 7) was used to examine the moderated mediating effect of perceived autonomy. The results showed that the interaction effect of perceived busyness and perceived autonomy on the sense of control was significant [Effect = −0.035, SE = 0.012, 95% CI: (−0.059, −0.012)], indicating that perceived autonomy significantly moderated the effect of perceived busyness on the sense of control. Simple slope analysis further revealed (see Figure 7) that at a low level of perceived autonomy, as the level of perceived busyness increased, the lack of sense of control became more severe [Effect = 0.513, SE = 0.231, 95% CI: (0.056, 0.970)]. At a high level of perceived autonomy, as the level of perceived busyness increased, the lack of sense of control decreased [Effect = −0.477, SE = 0.228, 95% CI: (−0.928, −0.026)]. Moreover, the moderated mediation effect of perceived autonomy was significant [Effect = −0.029, SE = 0.012, 95% CI: (−0.057, −0.008)], and further analysis of the data found that under the condition of low perceived autonomy, the influence of perceived busyness on consumers’ nostalgic consumption intention through sense of control was positively significant [Effect = 0.423, SE = 0.238, 95% CI: (0.025, 0.954)]. Under the condition of high perceived autonomy, the influence of perceived busyness on consumers’ nostalgic consumption intention through sense of control was negatively significant [Effect = −0.394, SE = 0.198, 95% CI: (−0.814, −0.043)].

#### 3.4.3 Discussion

Study 4 demonstrated the moderated mediation effect of perceived autonomy. Specifically, when consumers have a high level of perceived autonomy, the lack of control caused by a sense of busyness is weakened, which in turn reduces consumers’ nostalgic consumption intention. When consumers have a low level of perceived autonomy, the lack of control caused by perceived busyness is intensified, which in turn increases consumers’ nostalgic consumption intention.

## 4 Conclusion and discussion

In the fast-paced life of the 21st century, perceived busyness has become a widespread global phenomenon. The time pressure and heavy tasks that accompany perceived busyness often lead to negative psychological reactions, profoundly affecting people’s mental health. Against this backdrop, the global nostalgic consumption market is growing at an astonishing rate, with an expected scale of $200 billion by 2027 and an annual growth rate exceeding 8% (data from MarketResearch.com). As consumption serves as an important indicator of residents’ wellbeing, the prevalence of nostalgic consumption reflects the increasing demand for psychological homeostasis in this rapidly changing era.

This study utilized representative Chinese data to systematically explore, through four experiments, the impact of perceived busyness on nostalgic consumption intention and its underlying psychological mechanisms. The research findings indicate that perceived busyness significantly enhances individuals’ preference for nostalgic consumption, and this relationship is mediated by the deprivation of a psychological sense of control. Furthermore, individuals’ perceived autonomy plays a crucial moderating role: when individuals perceive a high level of autonomy, the driving effect of perceived busyness on nostalgic consumption through a lack of control is significantly diminished.

### 4.1 Theoretical implications

Our research findings support existing literature suggesting that consumer behavior, as a compensatory control mechanism, can restore individuals’ normal perception of their sense of control (Beck et al., 2020; Chen et al., 2017; Huang et al., 2023; Landau et al., 2015). These discoveries deepen our understanding of consumer psychology and behavior under the perceived busyness and expand the scope of relevant theoretical research. Firstly, this study enriches research on perceived busyness. Existing studies on perceived busyness have primarily focused on its impact on individuals’ social cognition, physical health, and mental wellbeing, with relatively limited attention to consumer behavior. By linking the sense of busyness to consumer behavior, this study reveals the positive influence of perceived busyness on nostalgic consumption, thereby addressing the gap in previous research on busyness regarding consumer behavior.

Additionally, based on compensatory control theory, this study demonstrates that a lack of control is a more central and crucial psychological mechanism in the influence of perceived busyness on nostalgic consumption willingness. This finding extends the impact of perceived busyness from the realm of positive psychological perceptions to that of negative psychological perceptions, providing a new perspective for explaining consumption choices in busy states. It reveals a psychological adjustment process of compensatory control in response to the fast-paced and high-pressure modern life, thereby broadening the explanatory scope and practical applicability of the theory.

Furthermore, by integrating self-determination theory, this study refines the boundary conditions under which the compensatory mechanism takes effect. The research finds that not all individuals experiencing busyness will equally turn to nostalgic consumption. High perceived autonomy, as an intrinsic psychological resource, can buffer the loss of control and thereby weaken the impulse for compensatory consumption. This discovery advances motivation research from focusing on the types of motivation (intrinsic vs. extrinsic) (Froiland and Worrell, 2016; Taylor et al., 2014; Vansteenkiste et al., 2006) to examining how the “state” of motivation modulates the transformation pathway from stressful psychological mechanisms to behavioral outcomes. It suggests that cultivating individuals’ sense of autonomy may fundamentally enhance their psychological resilience in coping with daily stress and reduce reliance on passive compensatory strategies.

### 4.2 Practical implications

Notably, the findings of this study have implications for promoting individual mental health and wellbeing in high-pressure environments. In fast-paced social settings, organizations and policymakers can alleviate the negative psychological consequences of busyness by designing environments that grant individuals more choice and control, such as flexible work arrangements and participatory decision-making. Meanwhile, recognizing nostalgia as an intrinsic, low-cost psychological adjustment resource, it can be incorporated into stress management and mental health education programs to guide individuals in actively utilizing nostalgic emotions for self-regulation.

### 4.3 Limitations

However, this study still has certain limitations that warrant further exploration in future research. Firstly, this study primarily relies on scenario-based experiments. Future research could employ the experience sampling method to capture the dynamic associations between individuals’ perceptions of busyness and their consumption intentions or utilize digital trace data (such as interactions with nostalgic content on social media) for more ecologically valid validation. Secondly, this study focuses on the subjective perception of busyness. Future research could investigate whether busy perceptions of different valences (positive busyness vs. negative busyness) influence consumption behavior through distinct mechanisms. Although the sense of control is a core mediator, needs for meaning or cognitive closure may also be important parallel mechanisms worthy of future exploration. Finally, the findings of this study are primarily based on the unique economic and cultural context of China. The distinctive “diligence” ethic and reverence for the past in Chinese culture may shape individuals’ unique cognitive and emotional connections to “busyness” and “nostalgia,” and their specific impacts require cross-cultural comparative studies in the future.

In conclusion, from the perspective of compensatory control, this study reveals that the prevalent perceived busyness in modern life drives a preference for nostalgic consumption by threatening individuals’ sense of control. This not only deepens our understanding of how the perception of busyness influences nostalgic consumption willingness but also suggests that, alongside focusing on economic output, maintaining individuals’ sense of control and autonomy over their lives is crucial for enhancing the overall mental health and wellbeing of society.

## Data Availability

The datasets presented in this article are available upon request from the authors. Requests to access the datasets should be directed to zhuxiaoping@home.hpu.edu.cn.
